# Cultural Adaptation of the Caregiver TLC Program to Promote Latino Dementia Caregivers’ Wellbeing

**DOI:** 10.1093/geroni/igaf122.2850

**Published:** 2025-12-31

**Authors:** Maria Quiñones-Cordero, Julian Montoro-Rodriguez, Ann Bilbrey, Jennifer Ramsey, Bruno Kajiyama, Claudia Kajiyama, Roberto Velasquez, Dolores Gallagher-Thompson

**Affiliations:** University of Rochester School of Nursing, Rochester, New York, United States; The University of North Carolina at Charlotte, Charlotte, North Carolina, United States; Optimal Aging Center, Sunnyvale, California, United States; York Technical College, Rock Hill, South Carolina, United States; Photozig, Inc., Moffett Field, California, United States; Photozig, Moffett Field, California, United States; Southern Caregiver Resource Center, Chula Vista, California, United States; Stanford University, Stanford, California, United States

## Abstract

Latino dementia caregivers provide more intensive (time and level) care, experience worse mental health outcomes, and receive less family and social support than other racial/ethnic groups. However, few evidence-based, culturally attuned behavioral interventions exist for Latino caregivers, and none focus on targeting social connectedness to improve emotional well-being. This study collaborated with a Community Advisory Board (CAB) to culturally adapt the Caregiver Thrive, Learn, and Connect intervention for Latino dementia caregivers and identify strategies to improve caregivers’ social connectedness. The CAB included six Latino community leaders, serving caregivers from San Diego, Rochester, and Charlotte, who co-adapted the program following the Cultural Adaptation Behavioral Health Stage Model. We documented CAB feedback using the Framework for Reporting Adaptations and Modifications – Enhanced. CAB feedback centered on intervention content, examples, and improving social connectedness. We then conducted a Stage Ia trial (N = 5) and assessed program acceptability and satisfaction through focus groups. Data was analyzed using rapid qualitative analysis. CAB-recommended modifications included language considerations (e.g., “overwhelmed” vs. “burdened”), aligning social connectedness goals with cultural values, developing culturally relevant examples to teach skills, and condensing material to allow more time for skill-building exercises and discussions. Participant feedback from the Stage Ia trial was categorized into four themes: socially oriented activities, content augmentation, integrating self-care, and time allocation. All participants rated program satisfaction at 10/10. This collaborative adaptation process informed further refinement of the adapted intervention and a subsequent Ib early-stage trial to evaluate engagement of a social connectedness target and improvements in mental health outcomes.

